# Improving the treatment of people with dementia. An action research project in North Kivu Province, Democratic Republic of Congo

**DOI:** 10.4314/ahs.v25i2.34

**Published:** 2025-06

**Authors:** Geoff Harris, Heri Dunia

**Affiliations:** International Centre of Nonviolence, Durban University of Technology

**Keywords:** Dementia, North Kivu Province, Democratic Republic of Congo

## Abstract

The estimated number of Africans suffering from some form of dementia in 2015 was 2.13 million and this is projected to rise to 7.62 million by 2050. Many sufferers engage in irrational behaviour which, across many African countries, is interpreted as resulting from witchcraft. As a result, the families of dementia sufferers are ostracised and dementia sufferers themselves are subject to violence. This article reports an action research project carried out in four rural communities near Goma in the eastern Democratic Republic of Congo in 2022 and 2023. In the exploration phase, data was collected from the families of dementia sufferers and from other community members concerning their experiences, attitudes and behaviour towards dementia sufferers and their families. This provided the basis for an intervention in the form of a community education programme which presented objective information about the disease. An evaluation of the outcomes of the intervention in two of the communities was carried out nine months later and found that there had been a dramatic positive change in attitudes and behaviour towards dementia sufferers, both by their families and by community members in general.

## Introduction

The potential challenges of dementia in sub-Saharan Africa can be seen in the numbers of people with some form of this neurological disorder who it is estimated will increase from 2.13 million in 2015 to 7.62 million by 2050^1^. There are no official figures concerning the prevalence of dementia in the Democratic Republic of Congo (DRC) but applying the prevalence estimates of 6.2 per cent for people aged 65 and above in and around Kinshasa^2^ to the 3.7 million people in that age group countrywide (UNFPA, 2003) results in an estimate of 230 000.

Many parts of the country, particularly in the North Kivu, South Kivu and Ituri Provinces, have high levels of post-traumatic stress as a result of extreme forms of violence and the displacement which has affected much of the population since the mid-1990s^4^. An area of Ituri Province which had gone through significant displacement since 2017 and violent clashes in 2020 had significant levels of psychological distress in 85 per cent of its population^5^. There is increasing evidence that high levels of trauma can be an important risk factor for dementia^6^. There is very limited capacity in the DRC to treat or care for those with mental illnesses and dementia. Almost all people with mental illnesses and neurological disorders such as dementia are cared for by their families, often kindly but sometimes harshly, given the burdens placed on caregivers.

Across Africa, illness and disability is frequently interpreted as resulting from the action of evil spirits and witchcraft and this is true for unusual behaviours resulting from mental illness. Violence against people with dementia and the ostracism of their families is a common community response^7^, often at the behest of pastors and prophets from revivalist and syncretic churches who also earn money from ceremonies aimed at casting out demons^8^. This violence ‘run[s] counter to customs and traditions that … [led to older people] being loved, protected, respected … Now the older people are subject to stigma, rejection, and sometimes physical or psychological abuse’^9^.

Hardly any of the very few studies of dementia the DRC have been conducted in the eastern provinces. Accordingly, the specific objectives of the project reported in this article were

1. To explore the knowledge of dementia in several communities in North Kivu Province and the treatment of people with dementia
2. Based on the exploration, to plan and carry out an intervention aimed at improving the treatment of people with dementia by community members
3. To evaluate the short-term outcomes of the intervention.

## Context

The role and capacity of government in areas such as education and health has progressively declined as a result of civil wars which have plagued North Kivu Province in particular, but also the adjacent South Kivu and Ituri Provinces. There has been heavy loss of life from battle deaths and the killing of civilians but more particularly as a result of food shortages, the collapse of health services and very large numbers of internally displaced people. During the first eight months of 2023, an estimated 1.5 million people in the three provinces were displaced, bringing their total to 5.8 million and to 6.9 million for the DRC as a whole^10^. Many families and whole communities have been displaced on multiple occasions.

Four communities between 15 and 35 km north of the provincial capital, Goma, were identified as being reasonably typical of rural communities in the province. The communities - Lac Vert, Mugunga, Nzulo and Kimoka – have populations ranging between 400 and 600 and are accessible by motorbike from National Road 2 leading north to Masisi and beyond. The first two are made up of people displaced by communal violence in Masisi, some 80 km to the north, during the 1990s. These people regard themselves as homeless and refugees, with very limited access to land. Each community is heavily dependent on subsistence farming to provide their basic needs and the two adjacent to lakes supplement this with fishing; some produce is sold.

There is virtually no provision of government services. Churches play an important role in each community and an association called Sauti ya Wazee, a Swahili term meaning ‘The voice of the old people’, which advocates for access to land and medical care for older people, has been formed in Lac Vert and Mugunga. Given the limited impact of government throughout the entire country, it is not surprising that that the association has had limited success to date but, as we shall see, it came to play an important role in this research project.

## Research methods

The research was based on the action research approach originally proposed by Kurt Lewin^11^. In essence, Lewin argued that research which leads to an understanding of the various dimensions of a social problem is not of itself likely to bring about change. He proposed extending this diagnostic or exploratory phase of research with an intervention specifically designed to bring about change. He also emphasised the importance of participation; that is, researchers working with those affected in order to bring about change, rather than imposing their own ideas and solutions.

Standard texts on action research^12^, typically identify four stages – exploration, planning an intervention, implementing the intervention and evaluating its outcomes. Subsequently, there is the possibility of planning and implementing a modified intervention. A recent article^13^ has reviewed five recent studies from Africa, using action research.

The exploration phase took place between the start of March 2022 and mid-June 2022. Having first received permission from community leaders, a snowball method was used to select 10 households which had a member thought by other household members to be showing signs of dementia. The main carer in each household was asked about their understanding of dementia, the work involved in caring, the responses of other community members and the support they received from elsewhere. Supplementary data was gathered from focus group discussions (FGDs) of ten adults aged 35-64 years held in each community, the members of which were chosen by community leaders or, in Lac Vert and Mugunga, by Sauti ya Wazee. There was a high degree of interest in these FGDs and many other community members listened in while they were being held. In addition, semi-structured interviews were held with 14 key informants comprising health practitioners, church leaders, community leaders and NGO workers.

In the intervention phase, data from the exploration phase was used along with medical data and with the participation of community members to draw up a programme to educate community members about dementia. The intervention was similar to that used in a project with rural communities in Lesotho^14^, and involved a training session of around two hours in each community during November and December 2022, led by the second author.

The short-term outcomes of the intervention were evaluated in two of the four communities (Lac Vert and Mugunga) in late August 2023. The other two communities could not be accessed due to fighting in their vicinity between M23 rebels and Congolese government forces which was continuing as of late 2023.

Permission to conduct the research was granted by the provincial government and the respective communities. Ethical approval was granted by Durban University of Technology's Institutional Research Ethics Committee (IREC 016/22, 18 February 2022) and we followed their protocols strictly, including informed consent, voluntary participation, anonymity and minimising risk to people with dementia.

## Results

### Exploration

The words of participants and informants quoted below represent commonly held views, unless otherwise stated.

### Awareness and knowledge of dementia

People in the four communities are aware of a progression of symptoms on the part of many older people which they termed Malali ya Buzehe or Magonjwa ya Wazee in Swahili, that is ‘the old people's disease’. Forgetfulness is the starting point. ‘My mother-in-law forgets things around her, she does not want to eat everything that we do and when she takes a walk in the area she forgets where the house is’ (Caregiver interview, Kimoka). This progresses towards increasing dependency as a result of a loss of strength by older people and their subsequent inability to look after themselves. In some older people, this progresses to irrational and/or antisocial behaviour which can lead to harsh responses by community members.

### Causes of dementia

In the interviews and FGDs, three levels of causal factors were commonly identified. This first was age, with participants being clear that most old people would almost inevitably go through the progression of symptoms mentioned above. The second was ‘excessive thinking’ which followed stressful event which led to anxiety and then to depression. As a caregiver from Lac Vert explained, ‘[My brother's sickness] followed the death of our father. Then came the expropriation of the plots of land we had. From there my brother started to develop seizures and loss of memory [as a result of] too much thinking’. The idea of ‘excessive thinking’ was often linked to worrying about survival as older people became less able to grow their own food or earn income to enable them to buy food. ‘Stress stems from too much thinking about daily hardship. Then people get caught up into a vicious circle of thinking about the problems surrounding them and end up developing the old people's disease’ (Caregiver, Kimoka).

Third, any strange behaviour on the part of older people – whether irrational or antisocial - is liable to be explained as being the result of demon-possession or of being a witch. For many community members, there seemed to be only a small gap between an older person losing some physical and mental capacities and being labelled as demon-possessed or a witch. Physical violence against such older people can take the form of stoning by adolescent males and being beaten by mobs, often during the night, with the aim of driving them out of the community.

### Caring for older people

The majority of older people continue to live with their families, with daughters-in-law often shouldering much of the burden of care. The following is typical of the comments made by caregivers: “Looking after an older person is not at all easy. They are often short tempered. They sometimes want to kill themselves. They want to leave when something goes wrong, They are forgetful. They claim more food and want particular types of food and they cry if you don't give it to them. They want to change family plans. They are depressed when they think of who they were and what they have now become. They need not just care, but intense care.” (Caregiver, Kimoka) As the demands on caregivers increase as dementia progresses, some older people are abandoned because their families cannot afford to provide the needed care and because of intense pressures from other community members to control the behaviour of their older relative.

As far as the community is concerned, three comments were commonly made. First, there is a good deal of caring activity by community members during the early stages of dementia: ‘If a grandpa or granny is lost, a person who comes across him/her normally asks people in the area to identify them in order to bring them back home’ (Community member, Nzulo). Neighbours often check on the older people who live alone to comfort them and keep an eye on them. Second, ‘old people are not interesting anymore’ (Community member, Lac Vert) and are therefore often ignored. As one said, ‘I'm alone most of the time. Who wants to stick around old people nowadays?’ Community members are busy about the various responsibilities and demands of life and choose not to do the time-consuming work of looking after older people.

A third response is active rejection of older people and sometimes their physical separation. The community of Kimoka, for example, is known as a place where the majority of the population are older people who have been placed there by their families, or who have been driven out of their own communities after accusations of witchcraft or who have somehow gravitated there because there is no one able to care for them elsewhere.

Their relegation to ‘a place of the unwanted’ stresses and depresses older people and they feel more and more rejected and useless. Overall, the traditional respect for older people has largely vanished and the once common experience of them ‘seeing out their days on their farms in the care of their children’ is now the exception. Staff at the hospitals and clinics below the rank of fully-trained doctors have virtually no medical knowledge of dementia and very little to offer people with dementia in terms of treatment. In addition, they typically operate on the principle that medicines are for younger people and not older people who will presumably die in the near future. ‘We [older women] are told there is no medication for the old people here and to go back home. It is as if we are being told to go away and die at home’ (Older woman, Nzulo). A nurse from a government clinic confirmed this practice: ‘When one is over 70 years old, he is denied healthcare at the hospital. Medicine is not for old people. It is for those who are still young and vibrant’. Apart from treatment for HIV-AIDS, tuberculosis and cholera or child vaccinations, where the general public is treated for free, treatment requires payment of fees which most of the study population cannot afford. The remaining alternative is to go to a chemist where they can buy basic generic medicines.

In a context where help for older people from the state is close to non-existent, faith communities are important in filling the gap, particularly for those who belong to a local congregation. This support often begins with prayer and counselling and, with some churches, prayers for the casting out of demons in cases where Malali ya Buzehe has been ‘diagnosed’. Some Catholic parishes in particular have specific programmes to help older people with some basic needs.

The main insights from the exploration component of the study can be summarised as follows: First, dementia is only one of a number of health challenges facing older people, with high blood pressure, sugar diabetes and deteriorating eyesight being those most often mentioned by health practitioners. Its presence was not measured directly but is clearly far more common than its estimated incidence in Kinshasa. It is likely that dementia is intertwined with post-traumatic stress disorder resulting from on-going violence and displacement since the mid-1990s. Second, given the very limited health facilities provided by the state, the burden of caring for older people with dementia falls on their families, a burden which increases as people with dementia become increasingly dependent. Most other community members are at best indifferent and at worst act violently against those older people who show any signs of dementia. The latter is based on fear of the unusual and ignorance regarding dementia. In response to these findings, we planned and implemented an intervention in the hope of increasing knowledge of dementia and encouraging support for people with dementia and their families.

### Intervention

The intervention involved the second author, together with a lecturer and two students from the Department of Health Studies at sub-Saharan Africa University in Goma. It involved a community-education session of around two hours in each of the four communities during December 2022, which were built around the following issues:

The meaning of dementia, its likely incidence in older people, its progression and its non-curability;
the causes of dementia, with particular reference to the role of witchcraft;
the needs of people living with dementia; and
The needs of those caring for those living with dementia.

### Short-term outcomes

The evaluation took place in August 2023 in Lake Vert, based on a FGD with 10 participants of varying ages from Lac Vert and Mugunga. Fighting in the vicinity of the other two communities made it impossible to carry out an evaluation there. In addition, the turnout from Lac Vert and Mugunga was lower than expected because of the proximity of the Bulengo camp for people displaced by violence perpetrated by the M23 rebel movement. This camp sprang up spontaneously in early 2023 and by March had over 100 000 residents living in tents and provided with food and other necessities by international relief agencies^15^. Between the times of the exploration and evaluation phases, many of those living in the four communities moved to Bulengo in order to benefit from the humanitarian assistance. These included members of the “Sauti ya Wazee”.

The first outcome of the training, according to the FGD participants, was an understanding of dementia which did not previously exist. The second was a dramatic change in attitudes and behaviour towards people with dementia, away from indifference and fear towards friendship and support. A significant factor in these positive changes was the spontaneous involvement of the Third Age Club members who were so impressed by the content of the training that they organized a follow up team to spread their newfound knowledge: “We have seen tremendous change taking place in our community. Those with parents with dementia have been told about the training and have started to look after their loved ones better. To give just one example, once the people of the Third Age Club passed by his home to share the teaching about caring for people with dementia, one adult son began to pay rent to his parent in whose house he was living.” (Older male FGD participant) Youth who attended the training in December 2022 also became involved in passing on information about dementia across the four communities. As a result, the message about dementia was spread across communities. The FGD estimated that three quarters of residents were now able to distinguish between what dementia is in reality as opposed to relying on an uncaring and violent tradition. “We have come to the point where people understand that dementia will affect many older people. The youth have helped us create that awareness and spread the message widely. Those who did not attend the training have nonetheless heard about and serious changes have taken place. Having gained a better understanding of the issues around dementia, we see youth helping people with dementia.” (Older female FGD participant)

Participants agreed that there had been a change in attitude towards older people in general.

You see, old age has regained some of its traditional value. After the campaign that the Third Age Club initiated after the training, we have seen old people being understood as people who are getting old rather than as witches or mad people. They are now left to live peacefully in their houses and are assisted by family members who were sceptical of their worth only yesterday. Some dignity for those in the third age has been restored … (Older female FGD participant).

A referee asked whether there were any negative comments or outcomes following the intervention and its implications for caring for PWD and their families. i.e. whether there was resistance to the new understanding of dementia. It may well be that individuals with alternative viewpoints to those documented above were not part of the FGDs on which the evaluation was based and/or that they kept such viewpoints to themselves. Our experience, however, was a widespread and wholehearted acceptance of the message of the intervention.

## Conclusion

From the evidence reported above, it seems clear that a modest education intervention has brought about significant positive changes in the lives of people with dementia and their families. Residents in the four communities were willing to hear the message - that dementia will affect many older people who will need community support and assistance - and to change their behaviour accordingly. This outcome was much enhanced by the unanticipated support of the older person's organisation Sauti ya Wazee and also youth who did much to spread the message on a house-by-house basis. Also unanticipated and important were the improvement in the status of older people in general and the increase in social cohesion across the generations which flowed from the intervention.

But can such a simple intervention have such profound effects? We offer two pieces of supporting evidence. First, the results reported here are similar to those found for the related intervention in rural Lesotho. Second, the fact that there are such huge needs, so few resources and no outside support for many communities may well increase the likelihood that any support will be both welcomed and effective. The hugely positive outcomes of limited group psychosocial interventions in highly traumatised areas of Ituri Province^16^ provide evidence in support of this conjecture.

Finally, while the relocation of many residents from the four communities to Bulengo refugee camp hindered the research project reported here, it has provided an opportunity to replicate the intervention in the camp; this began in April 2024. In addition, planning has begun for a SSAU-led conference in 2025 bringing together government, civil society organisations, faith communities and universities to discuss ways of spreading awareness of dementia and the needs of people living with it. This is consistent with the work of the Dementia Friendly Communities initiative^17^.
